# Dental Implant Supported Restorations Improve Quality of Life in Osteoporotic Women

**DOI:** 10.1155/2015/451923

**Published:** 2015-06-03

**Authors:** Christine DeBaz, Jenna Hahn, Lisa Lang, Leena Palomo

**Affiliations:** ^1^Department of Periodontics, School of Dental Medicine, Case Western Reserve University, 2124 Abington Road, Cleveland, OH 44106, USA; ^2^Department of Comprehensive Care, School of Dental Medicine, Case Western Reserve University, 2124 Abington Road, Cleveland, OH 44106, USA

## Abstract

*Introduction*. The aim of this study is to compare the quality of life (QoL) in partially edentulous osteoporotic women who have missing teeth restored with dental implant retained restorations with those who do not and, secondarily, to report the rate of osteonecrosis in this sample. *Methods*. 237 participants completed the Utian QoL survey, a 23-question document measuring across psychosocial domains of well-being including occupational, health, emotional, and sexual domains which together contribute to an overall score. The subset of participants having dental implant supported prosthesis (64) was compared to the subset having nonimplant supported fixed restorations (47), the subset having nonimplant supported removable restorations (60), and the subset having no restoration of missing teeth (66). *Results*. ANOVA showed significant difference in all QoL domains between the four subsets (*p* < 0.05). Although 134 reported oral bisphosphonate and 51 reported IV bisphosphonate use, no signs of ONJ were identified in any participants. *Conclusion*. These findings show implant retained oral rehabilitation has a statistically significant impact over nonimplant and traditional fixed restorations, removable restorations, and no restoration of missing teeth in far reaching areas including occupational, health, emotional, sexual, and overall QoL. These findings also support future examination of psychosocial outcomes associated with oral rehabilitation and the incorporation of oral health into women's health promotion.

## 1. Introduction

As the proportion of people over the age of 55 increases in the United States, so does the uncertainty of age related changes which may impact dental treatment. One of these age related changes is osteoporosis. Osteoporosis is said to increase the risk of tooth loss in postmenopausal women, but the mechanism remains unclear [1]. Otherwise healthy, vibrant postmenopausal women take steps ranging from lifestyle changes (such as improved diet and exercise) to medications (hormone replacement therapy and bone sparing medications) to prevent and treat osteoporosis. Both the effects of postmenopausal osteoporosis and the risks associated with the medications used to treat it have complicated decision making during the planning phase of dental implant therapy [2–4]. Drugs from a class of bone sparing medications, the bisphosphonates, have been associated with a condition called osteonecrosis of the jaws (ONJ). Even though the incidence of ONJ is recognized to be less than 0.1%, there are reports of up to 24% of questionable cases. The cases are considered questionable because they have abnormal features relating to the jaws but no visible necrotic bone (pathognomonic of ONJ) such that the condition cannot be fully adjudicated [5].

Studies suggest that dental implant therapy improves esthetics and self-image [6, 7]. Although these dental implant studies did not focus on postmenopausal women, esthetics would seem an important parameter for this cohort. Investigations by cosmetic surgeons, which do focus on postmenopausal women, confirm that as women age the focus of esthetic procedures tends towards the lower third of their face as opposed to younger counterparts who focus on skin and nose [8].

Oral health related quality of life (QoL) in women is poorly understood. Traditionally, dental investigations are concerned with treatment efficacy involving clinical parameters rather than individual patient perspective. In contrast, The World Health Organization (WHO) recognizes the importance of oral-health-related QoL within this framework through campaigns which portray not only images of pain-free living but also aesthetic images using attractive smiles as an image of enhanced well-being [9]. As a result of this difference, recent research is refocused to consider how oral health affects far reaching aspects of life such as psychosocial interaction, self-esteem, intimacy, overall health, and performance at work [10].

In spite of the seemingly high importance of comfort, function, and esthetics in a growing cohort of postmenopausal women dealing with osteoporosis, the authors of this study could find no well-controlled investigation into the QoL outcomes related specifically to women with dental implants. The aim of this study is to compare the QoL in partially edentulous osteoporotic women who have missing teeth restored with dental implant retained restorations with those who do not. Secondarily, the aim is to report the rate of ONJ in the sample.

## 2. Methods

### 2.1. Ethical Requirements

The current study was approved by IRB (number 2014-814) in accordance with the Helsinki Declaration. All participants completed the study questionnaire freely and informed consent was implicit on agreeing to complete the written survey. This is an IRB approved retrospective observational study.

### 2.2. Participants

**Figure 1:**
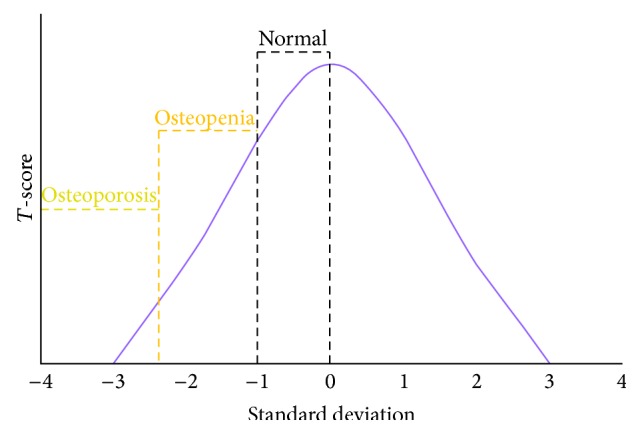
Standard distribution of *T*-score based on DEXA standard outcomes. Within 1 standard deviation of the normal healthy 30-year-old female standard, *T*-score of −1 is considered normal. *T*-score of −2 or two standard deviations less than the standard is considered osteopoenic, and *T*-score of −2.5 or less is considered osteoporotic.

**Figure 2:**
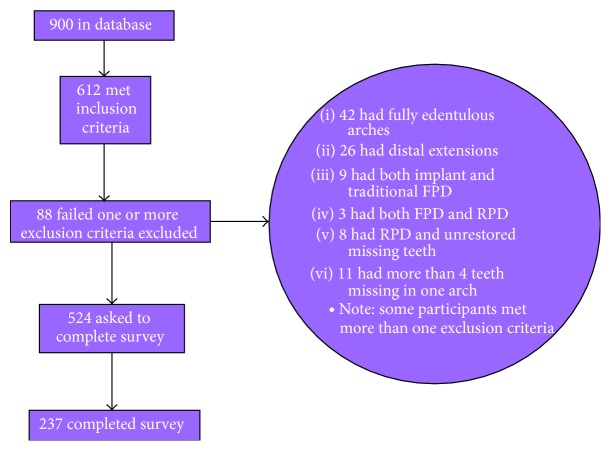
Study inclusion flowchart.

Participant charts were obtained from the Case/Cleveland Clinic Postmenopausal Wellness Collaboration (CCCPW) database of over 900 samples with dates of examination between January 2002 and October 2014 completed by trained panel of experts. The selection criteria included otherwise healthy, partially edentulous, postmenopausal women having osteoporosis. For this study, partially edentulous participant is defined as missing more than one tooth in adjacent sites, other than a premolars or third molars, at the time of examination for entry to the database. Menopause is defined by the absence of menses for at least 1 year either naturally or surgically. Osteoporosis is defined through Dual X-ray Absorptiometry (DEXA) scan *T*-score < −2.0 at either hip or spine within the last 5 years. DEXA is the medical diagnostic technique for measuring skeletal bone mineral density. It is taken at the hip or spine. The resulting measurement is a *T*-score. The score is the patient's bone density compared with a standard norm expected in a healthy 30-year-old woman. The *T*-score is the number of standard deviations away from the standard (Figure 1). Exclusion criteria included validated QOL confounders and are noted in the following list. After a complete CCCPW database search, 237 met inclusion, did not get excluded, and fully completed the survey (Figure 2). Ad hoc analysis shows that power of the study is 80% to detect a 2.1 difference in QoL outcome.

Exclusion criteria included the following:

 Greater than 4 missing teeth in one arch.
 Lost teeth currently under the process of replacement/rehabilitation.
 Fully edentulous arch with or without prosthesis.
 More than one type of restoration (implant supported, FPD, RPD).
 Distal extension RPDs.
 Systemic conditions associated with poor acceptance of oral prosthesis were as follows:
   Depression.
   Recreational drug use.
   Alcoholism.
   History of stroke/facial paralysis/Bell's palsy.

### 2.3. Clinical History

Pertinent patient medical history was recovered from the CCCPW chart generated by a panel of expert clinicians. Factors effecting inclusion/exclusion (including DEXA scores) were identified. BMI, use of tobacco, hormone replacement, and bone sparing medications were recorded. Presence/absence of clinical signs and symptoms of ONJ were likewise recorded. The ONJ condition is defined for the purpose of this study using the American Association of Oral Maxillofacial Surgeons updated definition. As such, patients may be considered to have ONJ if all of the following characteristics are present: (1) Current or previous treatment with antiresorptive or antiangiogenic agents, (2) exposed bone or bone that can be probed through an intraoral or extra oral fistula in the maxillofacial region that has persisted for greater than 8 weeks, (3) no history of radiation therapy to the jaws or obvious metastatic disease to the jaws [11]. As such, the definition of ONJ is based on exclusion. Clinical presentation of ONJ may take on a number of characteristics. If any of these characterizes cannot be traced back by the to a known etiology, (gingival abscess, endodontic failure, acute herpetic gingivostomatitis, etc.) then by exclusion the characteristics are said to be associated with ONJ (see the following list). 

*Clinical Features of Osteonecrosis of the Jaws (ONJ)*

 Symptoms may include the following:
   (a) Asymptomatic to numbness of pain
     (1) Limited to area around particular teeth.
     (2) Around alveolar bone.
     (3) Facial location.
   (b) Soft-tissue swelling.
   (c) Loosening of teeth.
   (d) Drainage.

### 2.4. Groupings

Participants who met inclusion, having dental implant supported prosthesis, were compared to the subset having nonimplant supported fixed restorations, the subset having nonimplant supported removable restorations, and the subset which had no restoration of missing teeth.

### 2.5. Utian Quality of Life Survey

Participants completed the Utian QoL survey, a 23-question document developed to fulfill healthcare providers' requests. It is a survey validated for postmenopausal women across socioeconomic and geographic areas. The questions contribute to a pool of items sampling across various aspects of well-being and include four basic domains: occupational, health, emotional, and sexual and together contribute to overall scores. Each question is answered on a scale of 1–5, where 5 is “very true of me” and 1 is “not true of me.” Mean scores were calculated for each item and domain and the overall summary score for each instrument was calculated.

### 2.6. Analysis

Participant characteristics were compared among the groups using the Kruskal-Wallis and Chi square tests. QoL outcomes of partially edentulous participants having implant restorations were compared to those who had fixed partial dentures, those who had removable partial dentures, and those who did not have any restoration using one-way ANOVA with threshold of significance at *p* < 0.05.

## 3. Results

Of the 900 participants in the database, 612 met the inclusion criteria. Of these, 88 participants were excluded due to the exclusion criteria. 237 fully completed the survey.

Table 1 notes participant demographics. Of the 237 participants, 64 had implant retained prosthetic restorations, 60 had traditional fixed partial dentures, 47 had removable partial denture, and 66 had no restoration to restore missing teeth. No significant difference in age exists between groups.

Table 2 shows the Utian QoL survey results for 4 separate domains including occupational, health, emotional, sexual, and overall scores.

One-way ANOVA analysis shows that there is a significant difference in each of the four domains measured in the Utian QoL survey and occupational, health, emotional, and sexual scores between the groups investigated. Additionally, there is a significant difference in the overall summary Utian QoL scores between the four groups.

## 4. Discussion

This investigation was initiated to incorporate oral health into women's health promotion and to examine psychosocial outcomes associated with dental implant supported rehabilitation [12]. This is the first study, as far as our authors could identify, on the subject of QoL focusing solely on postmenopausal women. This analysis compares the QoL in partially edentulous osteoporotic women who have missing teeth restored with dental implant retained restorations, nonimplant retained fixed restorations, removable partial dentures, and no restoration of missing teeth.

In the study of QoL, demographic and medical attributes have been argued as having an effect. This study focuses on Utian outcomes (occupational, health, emotional, sexual, and overall). For the purpose of complete reporting, Table 1 provides background demographic and medical data, even though statistical analysis of these background factors is beyond the scope of this study. In spite of no significant difference in age between groups, there is a significant difference in all QoL domains including occupational, health, emotional, sexual and overall QoL score between osteoporotic women who have missing teeth replaced by implant supported restorations, fixed partial denture, and removal partial denture and those with no replacement of missing teeth. Although our racial sampling is not sufficient to yield statistical significance, despite similarity in racial breakdown between groups, QoL is in all QoL outcome measures across the 4 groups. Similarly, tobacco use is widely believed to be a long term detriment to QoL; however, in spite of this, there is a significant difference in QoL outcomes in all categories measured, regardless of smoking history. Because current medical literature focuses on the QoL outcomes related to medications used for postmenopausal symptoms such as hormone replacement therapy and bone sparing medication use (oral and IV bisphosphonates, selective estrogen reuptake inhibitors, and RANKL inhibitors), use of these medications is included in the demographic patient population data.

No pathognomonic signs of ONJ, as spelled out in the most up to date position paper of the American Association of Oral and Maxillofacial Surgeons (AAOMS) [11], were identified in any of the participants. No clinical features, as presented in Clinical Features of ONJ list, and likewise no exposed bone of questionable origin were identified in the sampling. Of the 134 oral bisphosphonate users and 51 IV bisphosphonate users in our sampling, finding no ONJ case is within the expected incidence of 0.017%–0.1% reported by the American Society for Bone and Mineral Research and agreed upon by the American Dental Association and AAOMS position statement [13, 14].

These findings in postmenopausal women with osteoporosis support several widely held assertions revealed by previous investigations undertaken for the greater population. The first of these is that dental implants improve QoL and is shown in previous studies which compare QoL between pre- versus postoral rehabilitation [15–17]. Furthermore, literature suggests that tooth loss and reduced chewing ability are related to poor oral health QoL [18] and that dental implant supported tooth replacement improves QoL in improved comfort, speech, chewing function, and fit [19]. FPDs are considered superior to and have great patient acceptance than RPDs, poor diet, and unclear speech associated in the literature with RPD and edentulous patients overall may be responsible for this [20]. Although our findings support assertions put forth in previous studies, it is important to note that these previous studies are not limited to any one cohort. In fact, confounders such as age, gender, and Kennedy classification have been suggested. For the current investigation, distal extension RPD patients were excluded so as to remove this potential confounder; age is not significantly different between groups.

Interestingly, there is little difference between implant supported restorations versus fixed teeth supported restorations. Since fixed support structures support both of these, they confer a degree of stability to the prosthesis which is not found in other cases. Greater stability enhances comfort and function. Enhanced comfort and function may contribute to improved self-confidence. This self-confidence manifests in several areas of QoL.

Outside of investigations which compare pre- versus postrehabilitation around the benefits of implant supported restoration of missing teeth, QoL findings available in the literature are mixed. In fact, the current findings which employ a point in time comparison is in contrast with the results of a recent systematic review of 53 articles which concluded that although implant supported restoration outcomes were accompanied by high patient satisfaction related to comfort, bite force, and the ability to eat more tough foods, it did not translate to better QoL outcomes [21, 22]. Another recent, point in time study compared subjective QoL along with objective masticatory function in an attempt to generate a structural equation relating objective and subjective outcomes finds that perceived chewing ability is a critical factor for QoL and that masticatory performance rather than food mixing ability is important for perceived chewing ability and QoL. However that investigation was limited to RPD users and did not address either implant supported or traditional fixed restorations [23].

It is notable that influences of QoL, such as patient preferences, backgrounds, and interests, vary widely whereas singular measurements of chewing function, masticatory force, and fit, which may be more objective, do not [24]. Furthermore, multiple studies identify gender differences in prosthesis satisfaction [25, 26]. The current study focuses on women only to remove the potential confounding effect of gender. Our findings support Vogel et al., who in a review of literature evaluating cost-effectiveness of dental implant supported versus tooth supported fixed partial denture restorations found that, for multiple missing teeth, dental implants were associated with higher initial cost, but better improvements in oral health-related QoL versus other treatments [27]. Yet another review concludes that there is insufficient extractable information regarding the tooth versus implant supported prosthesis in a partially edentulous patient [28].

In light of inconclusive and contradictory review papers, we note that subjective patient-related factors and major determinants of QoL may be easier to investigate when the study is limited to specific cohorts. As such, focusing future QoL investigation on a more homogenous sample, such as ours, may be productive. Additionally, specifically validated instruments for homogenous cohorts, such as the Utian Survey in postmenopausal women, may be useful to study QoL. The use of a survey validated specifically for a target population separates the methodology used in the current study from previous ones. Previous studies used measurement instruments, such as Oral Health Impact Profile (OHIP), or a shorted version of that survey, which are generally applicable for generalizable groups.

## 5. Conclusion

In order to make decisions about the most appropriate treatment option in rehabilitation a dentist must understand not only the prosthetic therapeutic specifics such as chewing function and orofacial esthetics but also the patient-centered specifics of psychosocial and overall well-being. The results of the current investigation indicate that implant retained oral rehabilitation of missing teeth has a statistically significant impact over nonimplant and traditional fixed restorations, removable restorations, and no restoration of missing teeth in far reaching areas including occupational, health, emotional, sexual, and overall QoL. Within the limited diagnostic accuracy of the methods used for the identification of ONJ used in the present work, the incidence of bisphosphonate related ONJ seems to be very low and supports the rate cited in the most current literature.

## Figures and Tables

**Table 1:** Participant demographics. Characteristics of the study population (237 participants).

Factor	Implant *n* = 64	FPD *n* = 60	RPD *n* = 47	No restoration = 66
Age	62 ± 8.3	63 ± 7.4	59 ± 8.1	61 ± 10.9
Race *n* (%)				
White	31 (48)	27 (45)	26 (55)	32 (49)
Black	28 (43)	30 (50)	19 (40)	26 (39)
Hispanic	5 (8)	3 (5)	2 (4)	8 (12)
BMI				
<19	22	16	10	14
20–29	38	35	25	38
>30	4	9	12	14
Tobacco use				
Never	26	18	10	28
Former	34	30	18	25
Current	4	12	19	13
Hormone replacement				
Never	12	13	16	32
Former	39	35	26	22
Current	15	12	5	12
Bone sparing Medications				
Oral bisphosphonate (in the last 5 years)	35	32	31	36
Alendronate	24	22	18	20
Risedronate	5	6	10	12
Ibandronate	6	4	3	4
IV bisphosphonate (in the last 5 years)	12	15	10	14
Selective estrogen receptor modulators (SERM)	3	0	2	5
RANKL inhibitor	3	5	1	1
None	11	8	3	10

**Table 2:** Utian QoL survey results.

	Implant	FPD	RPD	No restoration	*P* value
Occupational score	26.79 ± 6.23	26.86 ± 5.11	21.42 ± 5.21	20.59 ± 3.56	<0.001
Health score	26.45 ± 6.30	21.32 ± 4.04	20.05 ± 4.89	19.23 ± 5.77	<0.001
Emotional score	25.75 ± 7.41	26.86 ± 6.05	17.03 ± 5.24	15.29 ± 4.99	<0.001
Sexual score	28.59 ± 8.57	24.84 ± 6.74	15.26 ± 4.65	11.45 ± 5.88	<0.001
Overall score	107.58 ± 7.25	99.88 ± 5.52	73.77 ± 5.02	66.56 ± 5.09	<0.001
